# Supplementary material for: Ancestral origins of TYR and OCA2 gene mutations in oculocutaneous albinism from two admixed populations in Colombia
Source: PLoS One. 2024 Nov 18;19(11):e0313777. doi: 10.1371/journal.pone.0313777 (PMC11573203; doi:10.1371/journal.pone.0313777)
Supplement: Attachment — Submitted filename: Ancestral origins of OCA2 study review.docx [file pone.0313777.s001.docx]

Ancestral origins of TYR and OCA2 gene mutations in Oculocutaneous albinism **2** from two admixed populations in Colombia**.**

The manuscript needs to be proof read with careful consideration to English language, grammar and sentence construction.

***Introduction –***

1. The Research study looks at the ancestral origins of 2 genes, mutations in which play an important role in manifestation of Oculocutaneous Albinism 2 (OCA2) in two admixed populations of Columbia.
2. The historical context about the region helps the reader understand the history of this region.
3. It is unclear from the present text as to why authors are interested in investigating mutations of TYR, and OCA2 genes. The research question needs to be better outlined. Elaborating this will explain the need to look at ancestral origins of OCA mutations.
4. This study builds on previous research done by the team, however inclusion of this data (in form of tables) needs to be incorporated and background work needs to be elaborated to build connections to present study (Page 5; 85-94; 106).
5. What are inbreeding estimates of the study populations? Authors should provide relevant data to support the statement that *“By examining population migration in the Andes regions and ancestral origins of the described mutations we report how both inbreeding and outbreeding increase the risk to develop autosomal recessive traits.” (Page 6; 111-113)*

***Results –***

6. Page 6; 116-118 – PCA analysis and global ancestry analysis should be explained for study populations.
7. The methods focus on haplotype comparisons using pedigree. The choice and justification of this methodological approach should be highlighted.
8. Results to be sectioned based on analysis. Detailed results to be explained for the pedigree and transmission of OCA mutations.
9. Role of inbreeding is not measured nor indicated in results as stated in Introduction

***Discussion –***

10. Page 8; 147-150 - This study is not investigating the complex demographic history of region but is trying to infer patterns of 2 gene mutations in the context of the complex demographic history of Columbia.
11. A large part of the discussion focuses on history and ancestry of these populations and region. While this is essential, authors should focus on genes under study and discuss their results in context of prior work and population history of the region.
12. Page 10; 181-183 – is there evidence of homozygosity due to inbreeding for other loci from this region to support this statement?
13. Page 10; 184-186 – Authors should provide reference of estimates of genetic diversity of *Bogotá*, the capital of Colombia.
14. Author’s proposal that compound heterozygotes found in individuals who carry mutations derived from varied ancestral sources can be due to outbreeding needs support from other population genetics studies. It may be useful to use population genetic models to explain how inbreeding and outbreeding affect compound heterozygotes.

***Methodology –***

15. Samples size is too limited to perform robust statistical analysis.
16. Methodological choice of taking pedigree data needs to be justified.
17. Authors should include a brief description of study participants and their recruitment here for clarity and not just cite the previous study.
18. How was the recruitment done for non-related individuals and what were ethical considerations while recruiting them.
