# Supplementary material for: Ancestral origins of TYR and OCA2 gene mutations in oculocutaneous albinism from two admixed populations in Colombia
Source: PLoS One. 2024 Nov 18;19(11):e0313777. doi: 10.1371/journal.pone.0313777 (PMC11573203; doi:10.1371/journal.pone.0313777)
Supplement: Attachment — Submitted filename: OCA_Admixed_Populations-ResponsestoReviewers.docx [file pone.0313777.s002.docx]

Editor comments and author responses

**Editor summary.** The study represents a small but curious study of genes influencing a Mendelian trait in admixed human populations. We had a hard time securing reviewers and the editor eventually took on the role of reviewing. We apologize for the delay. The work is of sufficient quality for PLOS one, but the MS lacks some polishing of narrative and the writing itself.

**Author response to editor summary.** We were pleased to read that editor and reviewer found our manuscript to be of interest and worthy of publication in PLOS ONE pending substantial improvements in the writing and narrative. We are particularly grateful that the editor took on the role of reviewing given the difficulty in securing reviewers. ***We had one of the native English-speaking coauthors (IKJ) completely rewrite the manuscript to improve the writing, focus, and narrative arc.*** We detail the changes made in response to the editor and reviewer comments below, indicating how and where we changed the manuscript in response to each point.

See the very excellent points by the reviewer, and the following from the editor.

The three major points are

**Editor comment # 1**. The results section lacks context and focus. Specifically, the second paragraph (Line 119.) dives straight to specifics and plenty of un-defined abberviations are used (IBS, NAM). Restructure this part, open with a general results statement that captures the details the follow. “First we analyzed a familial case, and then studied unrelated individuals…” Use the structure. We wanted to know … To address that we did … The data revealed … (rephrase as needed). See also minor points below

**Author response to editor comment #1.** We have revised the entire Results section as suggested to improve context and focus. We are sure to define all abbreviations when they first appear in the manuscript, but we have largely tried to avoid unnecessary abbreviations throughout (eg see revised Table 1). We appreciate the suggestions for how to structure this part, and we have tried to follow them throughout (see revised Results section).

**Editor comment #2.** The population genetic methods are lacking in detail. Starting Line 248. This includes how many samples analyzed? How many SNPs on this chip? How many polymorphic in your sample? How many markers / invidividuals were used in each analyses? Same dataset in all or subsets in some? Which version of MEGA? How wide a region around OCA/TYR?

**Author response to editor comment #2.** We have revised the entire Methods section as suggested to provide all of the key missing details as prompted by the editor (see revised Methods section with new subsections on Genetic variant analysis and Genetic ancestry inference).

**Editor comment #3.** The language needs considerable work. See numerous smaller pointers from the editor below, but you are strongly encouraged to scrutinize the manuscript for English grammar errors because many must have eluded me. Getting a 3rd party or native english friend to read may help.

**Author response to editor comment #3.** We are very grateful that the editor took the time and effort to provide so many suggestions for how to improve the grammar. ***As suggested, we had one of the native English-speaking coauthors completely rewrite the manuscript to improve the writing, focus, and narrative arc.*** Please note that instead of trying to improve the wording by addressing each of the editor’s very helpful grammar suggestions directly, we revised the entire manuscript. (Thus, we do not provide point-by-point responses to those comments).

**Reviewer #1 comments and author responses**

**Reviewer #1 summary.** Ancestral origins of TYR and OCA2 gene mutations in Oculocutaneous albinism 2 from two admixed populations in Colombia. The manuscript needs to be proof read with careful consideration to English language, grammar and sentence construction.

**Author response to editor summary.** As suggested by the reviewer and editor, ***we had one of the native English-speaking coauthors completely rewrite the manuscript with careful consideration to the English language, grammar, and sentence construction.*** We greatly appreciate the comprehensive and thoughtful comments provided by the reviewer. We have addressed all of the reviewer’s comments and revised our manuscript accordingly. Below, we indicated how and where we revised the manuscript in response to each comment.

**Introduction**

**Reviewer #1 comment #1.** The Research study looks at the ancestral origins of 2 genes, mutations in which play an important role in manifestation of Oculocutaneous Albinism 2 (OCA2) in two admixed populations of Columbia.

**Reviewer #1 comment #2.** The historical context about the region helps the reader understand the history of this region.

**Author response to reviewer #1 comments #1-2.** We are glad that the reviewer found our study on the genetics of Albinism in two admixed populations of Colombia to be of interest and appreciate the recognition that the historical context is helpful to the readers.

**Reviewer #1 comment #3.** It is unclear from the present text as to why authors are interested in investigating mutations of TYR, and OCA2 genes. The research question needs to be better outlined. Elaborating this will explain the need to look at ancestral origins of OCA mutations.

**Author response to reviewer #1 comment #3.** We have completely re-written the Introduction and Discussion to provide better context on why we are investigating mutations of TYR and OCA2 genes. As suggested, we have clearly specific our hypothesis and research objective in the revised manuscript.

**Reviewer #1 comment #4.** This study builds on previous research done by the team, however inclusion of this data (in form of tables) needs to be incorporated and background work needs to be elaborated to build connections to present study (Page 5; 85-94; 106).

**Author response to reviewer #1 comment #4.** As suggested, we have worked to clarify how the findings of our previous work on the genetics of OCA, and the work of others in the field, informs the current study. In particular, we clarify that OCA-causing mutations were characterized in previous studies, whereas we are inferring the ancestral origins of the haplotypes on which these mutations reside for our current study (see revised Introduction and Table 1 for mutations characterize from previous work together with ancestry inferences from this study).

**Reviewer #1 comment #5.** What are inbreeding estimates of the study populations? Authors should provide relevant data to support the statement that “By examining population migration in the Andes regions and ancestral origins of the described mutations we report how both inbreeding and outbreeding increase the risk to develop autosomal recessive traits.” (Page 6; 111-113)

**Author response to reviewer #1 comment #5.** We have simultaneously tried to tone down the language on inbreeding and outbreeding, while also providing explicit support for any assertions related to these concepts from our own findings (eg Figure 3 and Table 1) and previous studies. We do not discuss inbreeding or outbreeding in the revised Introduction. Rather, we only bring in these concepts in the Discussion as a theoretical framing of our results (see Discussion page 11 paragraph 3). We do provide a citation to a previous study on inbreeding coefficient in the Marinilla-Santuario region, but we also mention here that “However, given the relatively low sample size of two Marinilla-Santuario samples, and the lack of pedigree linking these samples, this conclusion is not as well supported as the G47D founder mutation.” (see Discussion, page 12, paragraph 2).

**Results**

**Reviewer #1 comment #6.** Page 6; 116-118 – PCA analysis and global ancestry analysis should be explained for study populations.

**Author response to reviewer #1 comment #6.** As suggested, we provide additional details on PCA in the Methods section on Genetic ancestry inference (see Methods, page 6, first paragraph) and the Results section on Genetic ancestry and admixture of Colombian OCA patients (see Results, page 6, second paragraph).

**Reviewer #1 comment #7.** The methods focus on haplotype comparisons using pedigree. The choice and justification of this methodological approach should be highlighted.

**Author response to reviewer #1 comment #7.** We have rewritten the Introduction, Methods, and Results sections to clarify that we use haplotype analysis in order to infer the ancestral origins of TYR and OCA2 mutations.

**Reviewer #1 comment #8.** Results to be sectioned based on analysis. Detailed results to be explained for the pedigree and transmission of OCA mutations.

**Author response to reviewer #1 comment #8.** As suggested, in the revised manuscript we clarify exactly what the pedigree allows us to observe, ie the inheritance patterns on TYR mutations together with their ancestral origins in the Results (see page 10, first paragraph) and in the Discussion (see page 11, third paragraph and page 12, third paragraph).

**Reviewer #1 comment #9.** Role of inbreeding is not measured nor indicated in results as stated in Introduction

**Author response to reviewer #1 comment #9.** We have simultaneously tried to tone down the language on inbreeding and outbreeding, while also providing explicit support for any assertions related to these concepts from our own findings (eg Figure 3 and Table 1) and previous studies. We do not discuss inbreeding or outbreeding in the revised Introduction. Rather, we only bring in these concepts in the Discussion as a theoretical framing of our results (see Discussion page 11 paragraph 3). We do provide a citation to a previous study on inbreeding coefficient in the Marinilla-Santuario region, but we also mention here that “However, given the relatively low sample size of two Marinilla-Santuario samples, and the lack of pedigree linking these samples, this conclusion is not as well supported as the G47D founder mutation.” (see Discussion, page 12, paragraph 2).

**Discussion**

**Reviewer #1 comment #10.** Page 8; 147-150 - This study is not investigating the complex demographic history of region but is trying to infer patterns of 2 gene mutations in the context of the complex demographic history of Columbia.

**Author response to reviewer #1 comment #10.** We agree. We have removed this statement and completely rewritten the Discussion to focus on what we actually did in the paper, ie analyze the ancestral origins of TYR and OCA2 mutations.

**Reviewer #1 comment #11.** A large part of the discussion focuses on history and ancestry of these populations and region. While this is essential, authors should focus on genes under study and discuss their results in context of prior work and population history of the region.

**Author response to reviewer #1 comment #11.** We agree. We have removed much of this unnecessary material from the revised Discussion, which now focuses on the genes under study and the ancestral origins of the mutations in the context of prior work and the population history of the region.

**Reviewer #1 comment #12.** Page 10; 181-183 – is there evidence of homozygosity due to inbreeding for other loci from this region to support this statement?

**Author response to reviewer #1 comment #12.** We do have evidence from the phylogeny in Figure 2 and the pedigree shown in Figure 3 that the same haplotype was inherited from both parents for two affected individuals, pointing to some degree of consanguinity. Nevertheless, as stated in response to other comments on this same point we have simultaneously tried to tone down the language on inbreeding and outbreeding, while also providing explicit support for any assertions related to these concepts from our own findings (eg Figure 3 and Table 1) and previous studies. We do not discuss inbreeding or outbreeding in the revised Introduction. Rather, we only bring in these concepts in the Discussion as a theoretical framing of our results (see Discussion page 11 paragraph 3). We do provide a citation to a previous study on inbreeding coefficient in the Marinilla-Santuario region, but we also mention here that “However, given the relatively low sample size of two Marinilla-Santuario samples, and the lack of pedigree linking these samples, this conclusion is not as well supported as the G47D founder mutation.” (see Discussion, page 12, paragraph 2).

**Reviewer #1 comment #13.** Page 10; 184-186 – Authors should provide reference of estimates of genetic diversity of Bogotá, the capital of Colombia.

**Author response to reviewer #1 comment #13.** We have eliminated any unsupported statements on genetic diversity as suggested. Please see our response to the above comment #12 as well.

**Reviewer #1 comment #14.** Author’s proposal that compound heterozygotes found in individuals who carry mutations derived from varied ancestral sources can be due to outbreeding needs support from other population genetics studies. It may be useful to use population genetic models to explain how inbreeding and outbreeding affect compound heterozygotes.

**Author response to reviewer #1 comment #14.** On the one hand, our revised manuscript now shows clear evidence of admixture-derived compound heterozygotes, and we hope that this now comes across much more clearly. On the other hand, as stated in response to previous comments, we have completely rewritten the Introduction and Discussion to tone down the discussion of inbreeding and outbreeding. We only mention this now in the Discussion where we have explicit support from our own results and we tie this directly to previous theoretical literature on the subject (see Discussion page 11 paragraph 3, and page 12 paragraphs 2 & 3).

**Methodology**

**Reviewer #1 comment #15.** Samples size is too limited to perform robust statistical analysis.

**Author response to reviewer #1 comment #15.** In our revised manuscript, we now clarify that our study aims to provide a descriptive analysis of the ancestral origins of the TRY and OCA2 mutations. Despite the low sample size of our study cohort, the large amount of genome-wide variant data that we characterized (~720k variants) and the large number of global reference samples that we used (515) provide sufficient resolution for us to achieve this objective. New details on these numbers can be found in the revised Methods section on Genetic ancestry inference (see page 6, paragraph 1).

**Reviewer #1 comment #16.** Methodological choice of taking pedigree data needs to be justified.

**Author response to reviewer #1 comment #16.** In the revised manuscript, we more clearly illustrate the utility of the pedigree to simultaneously illustrate the inheritance patterns of TYR mutations in a Colombian family and their ancestral origins (see Results, page 10, paragraph 1; Discussion, page 11, paragraph 3 and page 12, paragraph 3).

**Reviewer #1 comment #17.** Authors should include a brief description of study participants and their recruitment here for clarity and not just cite the previous study.

**Author response to reviewer #1 comment #17.** As suggested, we provide additional details on study participants and their recruitment in the revised Methods section on Study cohort and ethics (see page 4, paragraph 3 to page 5, paragraph 1)

**Reviewer #1 comment #18.** How was the recruitment done for non-related individuals and what were ethical considerations while recruiting them.

**Author response to reviewer #1 comment #18.** As suggested, we provide additional details on participant recruitment and all ethical considerations in the revised Methods section on Study cohort and ethics (see page 4, paragraph 3 to page 5, paragraph 1)
