# Supplementary material for: Ancestral origins of TYR and OCA2 gene mutations in oculocutaneous albinism from two admixed populations in Colombia
Source: PLoS One. 2024 Nov 18;19(11):e0313777. doi: 10.1371/journal.pone.0313777 (PMC11573203; doi:10.1371/journal.pone.0313777)
Supplement: Attachment — Submitted filename: Revision comments.docx [file pone.0313777.s003.docx]

Manuscript Title: Ancestral origins of TYR and OCA2 gene mutations in Oculocutaneous albinism **2** from two admixed populations in Colombia

Authors have made alterations to the manuscript based on reviewers suggestions. However, clarification and changes on points listed below are needed.

Introduction –

1. **“We are *interested in the relationship between genetic ancestry, admixture, and the presence of mutations that cause oculocutaneous albinism (OCA) in Colombian populations from the Andean region.”*** – Why? can authors state why this trait (and thereby genes OCA2 and TYR) is of interest to them? Is there a phenotypic association of significance in this population?
2. ***The specific objective of this study was to test our admixture-derived compound
   heterozygote hypothesis by characterizing the ancestral origins of TYR mutations from Altiplano Cundiboyacense and OCA2 mutations from the Marinilla-Santuario region in Antioquia”*** - The proposed hypothesis - admixture-derived compound heterozygote hypothesis has support from a previous study looking at the G47D mutation in OCA patients living in the Canary Islands and Puerto Rico and suggested to have Sephardic Jewish origin based on its high prevalence among Moroccan Jews living in Israel [23]. If the authors are retesting this hypothesis in the Andean population, they need to explain the changed/varied context that demands retesting of the hypothesis. It could be as simple as effect of these heterozygote mutations on this population.
3. **“We characterized the genome-wide patterns of genetic ancestry for OCA patients from Altiplano Cundiboyacense and Antioquia, confirming European and Native American admixture with little African ancestry.. Our results underscore the genetic complexity of Mendelian disease in admixed Latin American populations” –** this is a part of this study? The placement of the paragraph is odd as part of the introduction. It is suggested to include this section as part of the introduction to the discussion.

**Results** – **“The OCA1 patients studied here were sampled from in …. or near Altiplano Cundiboyacense, primarily in the departments of Cundinamarca and Boyacá (Figure 1A) …. genomic variant data from African, European, and Native American reference populations.” –** This content is repeated. It’s already included in Methods Study cohort section.

4. “**Principal component analysis (PCA) shows that the Colombian OCA patients studied here group closely together and fall between the…** **principal component 2
   (PC2; Figure 1B)** - statement can be changed to PCA was performed to evaluate… and PCA shows ….
5. “The pedigree in Figure 3 shows the inheritance patterns and ancestral origins for OCA1 causing
   *TYR* mutations…. – Firstly, acquisition of Pedigree data (why, how) doesn’t find any mention in the methods section. Pedigree data is crucial to testing the admixture-derived compound heterozygote hypothesis, it must be included in the Methods section.

Discussion – The discussion needs to rewritten. Authors should concentrate their narrative from generic to specific or vice versa. At the moment the text oscillates between broad ancestry estimates and genetic diversity of Columbian populations’ at large and Andean populations in specific. Results are not discussed in the sequence in which they are presented in Results section.

Authors can consider the following points to revise the discussion.

1. The discussion can begin with **“Our study on the genetic etiology of OCA in Colombia highlights the valuable insights that can be obtained from clinical genomics studies of Latin American populations characterized by significant genetic admixture…..Andean region, where our study populations live, is characterized by two-way European and Native American ancestry, including a previously hidden *Converso* ancestry component (Figure 1) [12].”**
2. Why the focus on OCA?
3. Summarize the key findings here and explain their patterns in context of admixture and ancestry of study populations and highlight ancestry profile of Columbians in general. Highlight previous studies on genetic etiology of OCA in Colombia characterized by significant genetic admixture and OCA (OCA1 and OCA2) frequencies in Columbian populations if any. If not, states there aren’t any other studies investigating this.
4. Explain Pedigree data and draw parallels with other diseases studied in these populations.
